# L-shaped association of triglyceride glucose-body mass index and self-rated mental health among the middle-aged and older adults: a national cohort study in China

**DOI:** 10.3389/fpubh.2025.1672881

**Published:** 2025-11-25

**Authors:** Yanqin Li, Qi Gao, Fan Luo, Yuxin Lin, Ruqi Xu, Pingping Li, Yuping Zhang, Jiao Liu, Hongrui Zhan, Licong Su

**Affiliations:** 1 Division of Nephrology, National Clinical Research Center for Kidney Disease, State Key Laboratory of Organ Failure Research, Nanfang Hospital, Southern Medical University, Guangzhou, China; 2 Department of Nephrology, Wuhan Fourth Hospital, Wuhan, Hubei, China; 3 Wuhan Clinical Research Center for Metabolic Chronic Kidney Disease, Wuhan, Hubei, China; 4 Division of Nephrology, People’s Hospital of Yangjiang Affiliated to Guangdong Medical University, Yangjiang, China; 5 Department of Rehabilitation, The Fifth Affiliated Hospital, Sun Yat-sen University, Zhuhai, Guangdong, China

**Keywords:** TyG-BMI, insulin resistance, mental health, CHNS, prospective cohort study

## Abstract

**Background:**

Previous studies have shown that the triglyceride glucose-body mass index (TyG-BMI) is associated with cardiovascular disease, stroke, and cognition. Its relationship with mental health remains underexplored. We aimed to investigate the association between TyG-BMI and mental health in Chinese adults.

**Methods:**

This study utilized data from the China Health and Nutrition Survey (CHNS), an ongoing longitudinal cohort. Participants aged ≥45 years who completed at least two survey rounds between 2009 and 2015 were included. The TyG index was calculated as ln [triglycerides (mg/dL) × fasting blood glucose (mg/dL) / 2]. BMI was calculated as weight (kg) divided by height squared (m^2^). The TyG-BMI was the product of the TyG index and BMI. Self-rated mental health was assessed using a composite score based on three CHNS questions regarding vitality, happiness, and optimism. Restricted cubic spline (RCS) curves and two-piecewise multivariable Cox hazard regression models, which were adjusted for sociodemographic, lifestyle, and cardiometabolic factors, were employed to explore the relationship between the TyG-BMI and self-rated mental health. Models were adjusted for sociodemographic, lifestyle, and cardiometabolic factors.

**Results:**

Among 2,951 participants (47.6% male, median age 56.0 [25th, 75th percentile: 51, 64] years), the median TyG-BMI was 204.3 [25th, 75th percentile: 179.6, 231.8]. Over a median follow-up of 6.0 [2.0, 6.1] years, 1,026 (34.8%) incident was identified poor self-rated mental health. RCS curves indicated an L-shaped association between TyG-BMI and self-rated mental health (*p* for non-linear = 0.033), with an inflection point of 204.3. Below this threshold, each 10-unit increase in TyG-BMI was associated with a 6% decrease in self-rated mental health risk (adjusted hazard ratio [aHR] = 0.94, 95% confidence interval [CI]: 0.90–0.99). Each 1-standard deviation (SD) increase corresponded to a 20% risk reduction (aHR = 0.80, 95% CI: 0.67–0.96). Above the threshold, no significant association was observed. Subgroup and sensitivity analyses yielded consistent results.

**Conclusion:**

This study revealed an L-shaped association between TyG-BMI and self-rated mental health in mentally healthy, middle-aged and older Chinese individuals. Our findings suggest that TyG-BMI may serve as an effective tool for enhancing the primary prevention of mental health.

## Introduction

Mental disorders, including depression, anxiety, and stress, are among the leading causes of global disease burden and disability, affecting individuals of all ages, particularly in early adulthood as well as among middle-aged and older adults (1). The World Health Organization (WHO) estimates a 13% increase in mental health conditions over the past decade (2), with 322 million people living with depression worldwide in 2017 (3). In China, the adjusted prevalence of mental disorders has been reported to reach 17.5% (4). Several meta-analyses have confirmed a significant increase in the prevalence of mental disorders during the COVID-19 pandemic (5, 6). However, the lack of reliable methods to accurately assess and predict a wide range of psychological problems—considering both technical and subjective factors—complicates the early identification of mental health risks (7). Previous studies have identified genetic and environmental factors, such as social-ecological influences like socioeconomic status (8), maternal infection (9), drug abuse (10), and metabolic abnormalities (11, 12) as significant contributors to the occurrence of mental disorders. Therefore, it is essential to identify additional modifiable risk factors and develop targeted interventions to improve mental health in vulnerable populations.

Growing evidence suggests that insulin resistance (IR) is a key indicator of metabolic dysregulation (13). The triglyceride-glucose (TyG) index, calculated from triglycerides (TG) and fasting blood glucose (FBG) (14), has been proposed as a surrogate marker for IR. Several studies have have linked the TyG index to depression (15), cognitive decline (16), and stroke (17). Recently, the triglyceride-glucose-body mass index (TyG-BMI) has emerged as a more robust marker for early IR detection compared to the TyG index, since it integrates three well-validated parameters for IR recognition: TG, FBG, and adiposity (18). However, existing research predominantly focuses on TyG-BMI’s associations with cardiovascular events (19), stroke (20), dementia (21), and all-cause mortality (22). Few studies have explored the TyG-BMI–mental health relationship (23–25), and existing ones are limited by cross-sectional designs (23), young adults (24), or premenopausal and postmenopausal women (25). Given China’s aging population, understanding mental health in middle-aged and older individuals is critical. Furthermore, while associations with clinically diagnosed mental disorders exist, the link with subjective, self-rated mental well-being in the general population remains less explored.

Therefore, utilizing data from the China Health and Nutrition Survey (CHNS) (26), the present study aims to extend the current literature by: (1) investigating the shape of the relationship between TyG-BMI and self-rated mental health using non-linear modeling techniques; (2) determining a specific threshold, if it exists, to facilitate risk stratification; and (3) providing insights that are immediately relevant to population-level mental health promotion rather than only clinical diagnosis.

## Methods

### Study design, population, and data source

The CHNS cohort provided the study population^1^ (26). This ongoing prospective cohort, established in 1989, includes a nationally representative sample covering 15 provinces and autonomous regions, representing 47% of China’s population (27). By 2015, 42,829 participants had been enrolled across 388 communities. Ten follow-up waves (1989–2015) collected harmonized data on demographics, socioeconomic status, diet, lifestyle (smoking/alcohol use), and clinical health indicators through trained personnel. The present analysis utilized data from the 2009, 2011, and 2015 waves—the only cycles that included the laboratory data essential for constructing the TyG-BMI index. The 2009 survey served as the baseline. The study focused on the associations between TyG-BMI and mental health among middle-aged and older adults (aged at least 45 years), as the psychological well-being measurements were specifically designed for this age group in the CHNS.

**Figure 1:**
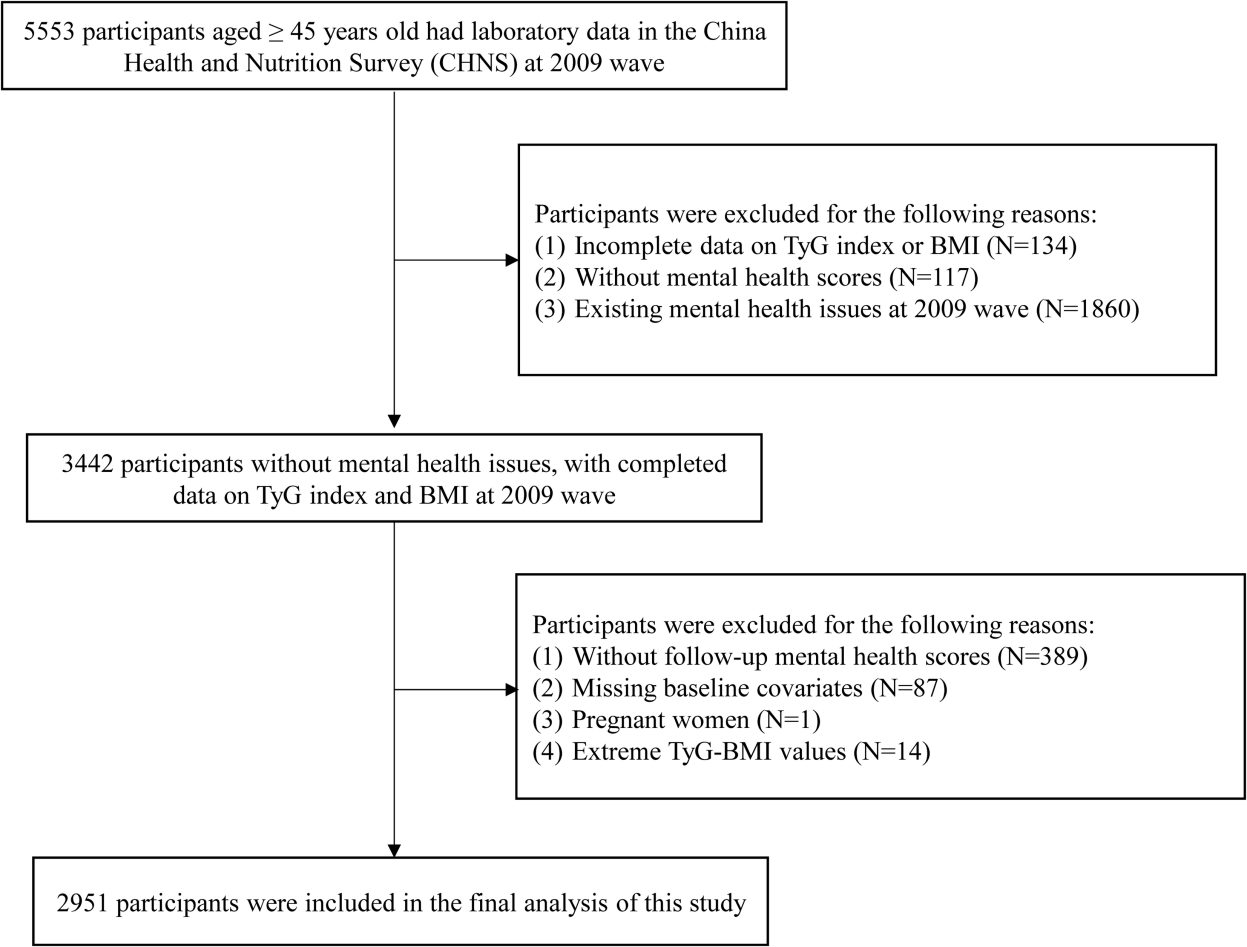
Flowchart of participants selection. *N*, number; TyG, triglyceride-glucose; BMI, body mass index.

Participants aged ≥45 years with complete laboratory data in 2009 (*N* = 5,553) were initially included. We excluded individuals without TyG index or BMI data (*N* = 134), those without mental health scores (*N* = 117), and those with pre-existing mental health issues in 2009 (*N* = 1,860). After these exclusions, 3,442 participants remained. Further exclusions were made for individuals without follow-up mental health scores (*N* = 389), baseline covariate data (*N* = 87, Supplementary Table 1), pregnant individuals (*N* = 1), and with extreme TyG-BMI values (more than or less than 3 standard deviation (SD) from the mean, *N* = 14). The use of a 3-SD cutoff is a conventional method for outlier removal, justified by the empirical rule, which states that approximately 99.7% of data in a normal distribution falls within 3 SD of the mean. Finally, 2,951 participants were included in the analysis (Figure 1).

The study was approved by institutional review boards at the University of North Carolina at Chapel Hill, the National Institute of Nutrition and Food Safety, and the Chinese Center for Disease Control and Prevention. All participants provided written informed consent. Reporting adhered to the STROBE (Strengthening the Reporting of Observational Studies in Epidemiology) guidelines (28) and the Declaration of Helsinki.

### Blood sample collections and measurements

Participants fasted for 8–12 h prior to venipuncture. Blood samples (12 mL) were analyzed at the China-Japan Friendship Hospital’s Ministry of Health laboratory. Total cholesterol (TC), triglycerides (TG), and high-density lipoprotein cholesterol (HDL-C) via enzymatic assays (Hitachi 7,600, Kyowa, Japan). Low-density lipoprotein cholesterol (LDL-C) was calculated using the Friedewald formula. Fasting blood glucose (FBG) via glucose oxidase method. The measurement methods for other biomarkers, including glycosylated hemoglobin (HbA1c), blood urea nitrogen (BUN), uric acid, hemoglobin, serum creatinine (SCr), high-sensitivity C-reactive protein (hsCRP), and fasting blood insulin (FBI), as detailed previously (27).

### Calculation of TyG-BMI

The TyG index was calculated using the formula ln [TG (mg/dL) × FBG (mg/dL) / 2] (14). BMI was calculated by dividing weight (kg) by the square of height (m). The TyG-BMI was derived using the formula ln [TG (mg/dL) × FBG (mg/dL) / 2] × BMI (18).

### Assessment of self-rated mental health

The study’s primary outcome was self-rated mental health status, assessed through the CHNS using three structured questions related to vitality, well-being, and optimism: “You have as much energy as you did last year,” “You are as happy as you were when you were younger,” and “Things are better than you think as you get older” (29). Responses were scored on a 5-point Likert scale (0 = “never” to 4 = “very often”). Total scores (range: 0–12) showed good internal consistency (Cronbach’s *α* = 0.801). Since there was no established cut-off point for mental health scales in the CHNS, the study used a mean self-rated mental health score of 9 from the analysis dataset as a reference (30). A score of less than 9 indicated no self-rated mental health (coded 0), while a score of 9 or greater indicated incident self-rated mental health (coded 1).

### Covariates

Participants provided demographic and socioeconomic data, including age, gender, residence (North/South), education (illiteracy/primary school/middle school/high school or above), marital status (married/unmarried/widowed/others), occupation (farmer/worker/unemployed/others), chronic diseases (yes/no), and smoking/alcohol habits (current/never and former). Trained staff recorded anthropometric data such as weight, height, waist, and hip circumference. The waist-to-hip ratio (WHR) was calculated as waist circumference (in meters) divided by hip circumference (in meters). Overweight was defined as a BMI > 24 kg/m^2^ in Chinese adults.

During follow-ups, trained staff measured seated blood pressure after a 5-min rest, using mercury sphygmomanometers with appropriately sized cuffs. Triplicate measurements were taken on the same arm, and the average systolic (SBP) and diastolic (DBP) pressures were used. Hypertension was defined as mean SBP ≥ 140 mmHg or DBP ≥ 90 mmHg, physician diagnosis, or antihypertensive use, according to WHO criteria. Diabetes mellitus (DM) was diagnosed based on physician report, use of hypoglycemic agents, fasting glucose ≥7.0 mmol/L, or HbA1c ≥ 6.5%, as per the American Diabetes Association (ADA) guidelines (31). Chronic kidney disease (CKD) was defined as an estimated glomerular filtration rate (eGFR) < 60 mL/min/1.73 m^2^, calculated using the Chronic Kidney Disease Epidemiology Collaboration (CKD-EPI) equation (32).

### Statistical analysis

Continuous variables were presented as mean ± SD or median [25th, 75th percentile] and compared using analysis of variance (ANOVA) or the Kruskal-Wallis test. Categorical variables were summarized as counts (*N*) and percentages (%) and compared using the chi-square test. Follow-up spanned from the 2009 baseline to the first occurrence of self-rated mental health, the 2015 survey, or loss to follow-up. Incidence rates were reported per 1,000 person-years.

Univariate and multivariable Cox proportional hazards models were used to estimate hazard ratio (HR) and 95% confidence interval (CI). Model 1 adjusted for age and sex; Model 2 further adjusted for WHR, smoking/alcohol use, geographic region, marital status, education, occupation, hypertension, CKD, DM, and biochemical markers (including LDL-C, TC, HbA1c, and FBI). The proportional hazards (PH) assumption was assessed using Schoenfeld residuals. The global test and the test specifically for TyG-BMI were non-significant (*p* > 0.05), indicating no violation of this critical assumption. All continuous covariates (age, WHR and biochemical markers) were modeled as continuous variables in the regression models. Variance inflation factor (VIF) (33) for all variables were <5, indicating no significant multicollinearity (Supplementary Figure 1). Restricted cubic spline (RCS) Cox regression, with 4 knots (5th, 35th, 65th, 95th percentiles) explored the dose–response relationship between TyG-BMI and self-rated mental health. The median TyG-BMI was used as the reference due to the lack of a standard (34, 35). A threshold analysis with two-piecewise Cox regression was conducted based on RCS results.

### Subgroup analyses

Associations were stratified by age (<65 vs. ≥65 years), sex (male vs. female), WHR (<0.85 vs. ≥0.85), SBP (<120 vs. 120–<140 mmHg), DBP (<80 vs. 80–<90 mmHg), smoking status (non-smoker vs. smoker), region (North vs. South), and presence of DM or hypertension (yes vs. no). Interaction terms tested effect modification.

### Sensitivity analysis

Three sensitivity analyses were performed. First, the standard Cox model assumes the exact time of an event is known. However, our study design only provided interval-censored data from survey waves. Hence, we applied an interval-censored Cox model, which is more appropriate for such data and provides less biased estimates (36). Second, we reanalyzed data from participants who had completed only two follow-up visits to determine whether the duration of follow-up potentially affected the outcomes. Third, to minimize the impact of missing values for specific biochemical indicators, we investigated the association between exposure and outcome using data imputed with the random forest method to ascertain whether using this method affected the primary results. The random forest method for imputation is capable of handling complex, non-linear relationships and interactions among variables without requiring specific assumptions about the data distribution (37). The random forest imputation was performed using the rfImpute function from the randomForest package in R. To ensure reproducibility, a random seed was set. The model was configured with 100 trees (ntree = 100) and 3 iterations (iter = 3). The model was constructed with the TyG-BMI index as the response variable (y) and a comprehensive set of predictor variables (x) that included all analysis variables.

### Additional analyses

E-value analysis was conducted to quantify potential unmeasured confounding (38). All analyses were performed using R software (version 4.4.1).^2^ Statistical significance was defined as two-sided *p* < 0.05.

## Results

### Study population and baseline characteristics

The flowchart of the study population selection is shown in Figure 1. The characteristics of included and excluded participants are summarized in Supplementary Table 2. The distribution of the TyG index, gender, DBP, WHR, smoking status, diabetes mellitus, and most laboratory data were similar between the two groups. However, a difference in BMI distribution was observed, leading to variation in TyG-BMI. Nevertheless, the 25th and 75th percentiles of TyG-BMI noverlapped nearly between the groups. Among the 2,951 participants selected for analysis (including 1,405 males [47.6%]), the median age was 56.0 years [25th, 75th percentile: 51, 64], with a median baseline TyG-BMI of 204.3 [179.6, 231.8]. Participants excluded from the dataset (*N* = 2,602) were older, with over 62% residing in the South. They also had higher rates of illiteracy and unemployment and were more likely to have CKD or hypertension.

The demographic and baseline characteristics of the included participants, stratified by the median of TyG-BMI, are summarized in Table 1. Generally, individuals in the higher TyG-BMI group were of similar age but had elevated levels of SBP, DBP, WHR, triglycerides, TC, LDL-C, HbA1c, FBG, and FBI compared to those in the lower group. They also had higher rates of DM and hypertension (all *p* < 0.05). Additionally, the higher TyG-BMI group had lower levels of HDL-C and a lower prevalence of smoking (all *p* < 0.05).

**Table 1:** Baseline characteristics of participants stratified by TyG-BMI index.

Characteristics	Overall (*N* = 2,951)	TyG-BMI		*p* value
		< 204.3 (*N* = 1,476)	≥204.3 (*N* = 1,475)	
TyG index	8.70 [8.20, 9.10]	8.30 [8.00, 8.60]	9.00 [8.70, 9.50]	<0.001
BMI, kg/m^2^	23.60 [21.40, 25.80]	21.45 [20.00, 22.70]	25.70 [24.40, 27.50]	<0.001
Age, years	56.00 [51.00, 64.00]	56.00 [51.00, 64.00]	57.00 [51.00, 64.00]	0.875
Male (%)	1,405 (47.6)	723 (49.0)	682 (46.2)	0.145
SBP, mmHg	127.00 [118.00, 140.00]	121.00 [112.00, 135.00]	130.00 [120.00, 144.00]	<0.001
DBP, mmHg	81.00 [76.00, 89.00]	80.00 [72.00, 86.00]	82.00 [79.00, 90.00]	<0.001
WHR	0.90 [0.80, 0.90]	0.90 [0.80, 0.90]	0.90 [0.90, 0.90]	<0.001
Smoking, *n* (%)	846 (28.7)	470 (31.8)	376 (25.5)	<0.001
Drinking, *n* (%)	983 (33.3)	495 (33.5)	488 (33.1)	0.825
Region^*^, *n* (%)				0.011
North	1,373 (46.5)	602 (40.8)	771 (52.3)	
South	1,578 (53.5)	874 (59.2)	704 (47.7)	
Education, *n* (%)				0.016
Illiteracy	829 (28.1)	431 (29.2)	398 (27.0)	
Primary school	601 (20.4)	302 (20.5)	299 (20.3)	
Middle school	1,224 (41.5)	620 (42.0)	604 (40.9)	
High school or above	297 (10.1)	123 (8.3)	174 (11.8)	
Occupation, *n* (%)				<0.001
Farmer	808 (27.4)	484 (32.8)	324 (22.0)	
Worker	99 (3.4)	58 (3.9)	41 (2.8)	
Unemployed	1,445 (49.0)	670 (45.5)	775 (52.6)	
Others	594 (20.2)	262 (17.8)	332 (22.6)	
Marital status				0.778
Married	14 (0.5)	8 (0.5)	6 (0.4)	
Unmarried	2,648 (89.7)	1,320 (89.4)	1,328 (90.0)	
Widowed	243 (8.2)	122 (8.3)	121 (8.2)	
Others	46 (1.6)	26 (1.8)	20 (1.4)	
CKD, *n* (%)	396 (13.4)	198 (13.4)	198 (13.4)	1
Diabetes mellitus, *n* (%)	398 (13.5)	106 (7.2)	292 (19.8)	<0.001
Hypertension	1,142 (38.7)	421 (28.5)	721 (48.9)	<0.001
Laboratory data				
Triglycerides, mmol/L	1.34 [0.92, 2.06]	1.00 [0.74, 1.36]	1.89 [1.34, 2.81]	<0.001
TC, mmol/L	4.95 [4.35, 5.65]	4.75 [4.21, 5.39]	5.18 [4.53, 5.86]	<0.001
LDL-C, mmol/L	3.07 [2.49, 3.69]	2.97 [2.46, 3.55]	3.18 [2.55, 3.87]	<0.001
HDL-C, mmol/L	1.40 [1.17, 1.66]	1.52 [1.30, 1.78]	1.26 [1.07, 1.50]	<0.001
HbA1c, %	5.60 [5.30, 6.00]	5.50 [5.20, 5.80]	5.70 [5.40, 6.10]	<0.001
FBG, mmol/L	5.26 [4.82, 5.77]	5.09 [4.67, 5.50]	5.48 [5.00, 6.11]	<0.001
FBI, mmol/L	10.32 [7.26, 14.96]	8.68 [6.39, 12.23]	12.39 [8.80, 17.89]	<0.001
eGFR, ml/min/1.73m^2^	75.00 [66.00, 84.00]	75.00 [66.00, 85.00]	74.00 [65.00, 84.00]	0.088

^*^Region was divided into north (Heilongjiang, Liaoning, Beijing, Shandong, and Henan), and south (Jiangsu, Shanghai, Hubei, Hunan, Chongqing, Guizhou, and Guangxi) based on the Qinling Mountains-Huaihe River Line.

*N*, number; TyG, triglyceride-glucose; SBP, systolic blood pressure; DBP, diastolic blood pressure; BMI, body mass index; WHR, waist hip ratio; CKD, chronic kidney disease; TC, total cholesterol; LDL-C, low density lipoprotein cholesterol; HDL-C, high density lipoprotein cholesterol; HbA1c, glycosylated hemoglobin A1c; FBG, fasting blood glucose; FBI, fasting blood insulin; eGFR, estimated glomerular filtration rate.

A total of 1,026 (34.8%) participants developed self-rated mental health during a median follow-up duration of 6.0 [2.0, 6.1] years. The baseline characteristics of participants stratified by self-rated mental health (yes/no) are compared in Supplementary Table 3.

### Association between TyG-BMI and self-rated mental health

**Figure 2:**
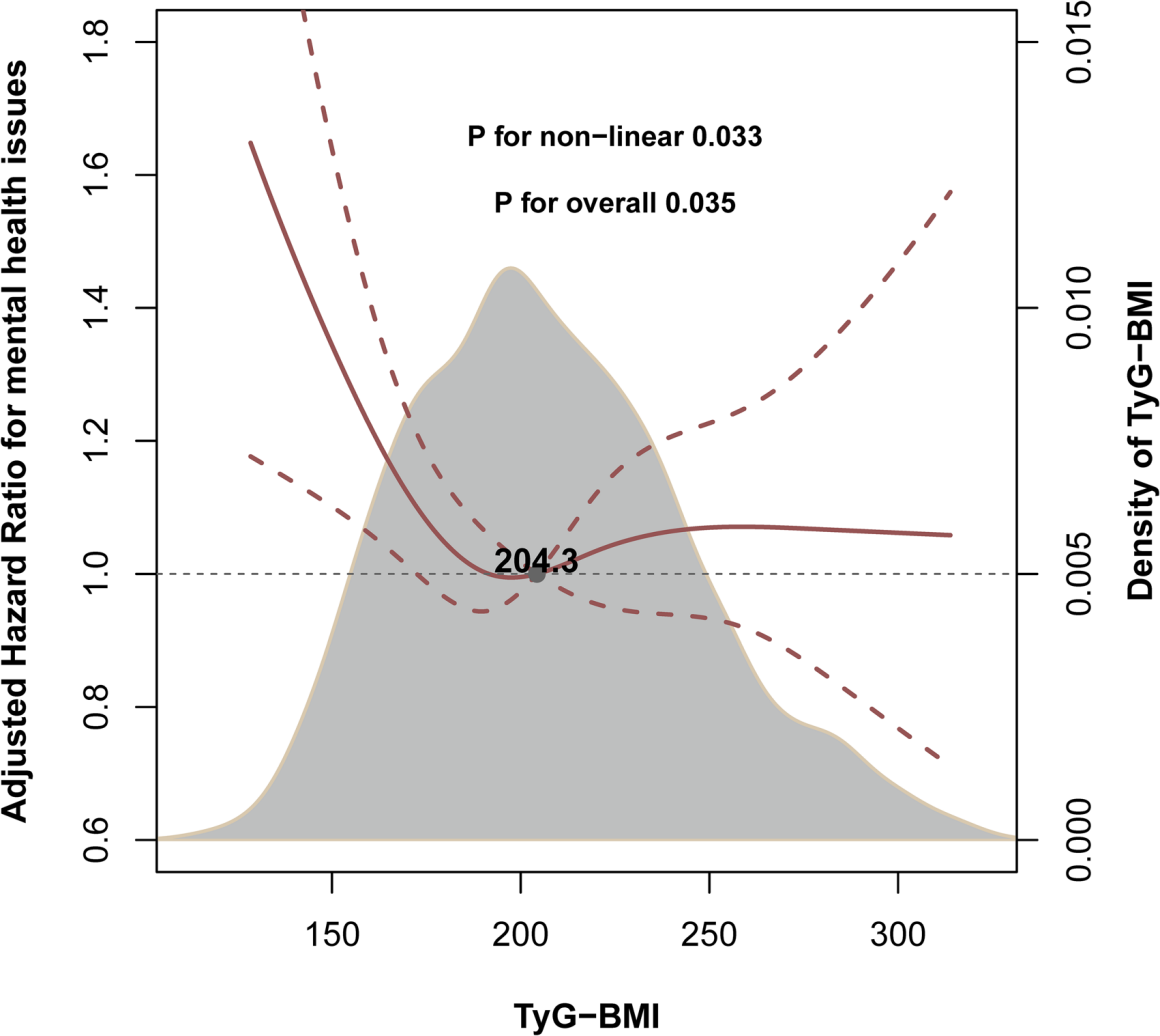
Levels of TyG-BMI and the risk of poor self-rated mental health. This figure depicts the smoothed curve from a restricted cubic spline model exploring the nonlinear relationship between the continuous TyG-BMI index and the risk of poor self-rated mental health. The solid line represents the hazard ratio (HR), and the shaded band represents the 95% confidence interval. The model was adjusted for sex, age, waist-to-hip ratio (WHR), smoking status, drinking status, region, marital status, education level, occupation, chronic kidney disease, hypertension, diabetes mellitus, low-density lipoprotein cholesterol (LDL-C), total cholesterol (TC), glycosylated hemoglobin A1c (HbA1c), and fasting blood insulin (FBI).

The crude incidence rates of self-rated mental health were 85.0 per 1,000 person-years for the TyG-BMI index <204.3 and 75.8 per 1,000 person-years for the TyG-BMI index ≥204.3. After adjusting for confounders, the Cox model demonstrated that in the group with TyG-BMI < 204.3, both a 10-unit increase and a 1 SD increase in TyG-BMI were significantly associated with a decreased risk of poor self-rated mental health (adjusted hazard ratio [aHR]: 0.94, 95% CI: 0.90–0.99 and aHR: 0.80, 95% CI: 0.67–0.96, respectively). Conversely, in the group with TyG-BMI ≥ 204.3, neither the 10-unit increase nor the 1 SD increase in TyG-BMI showed a statistically significant difference (Table 2). The dose–response curve indicated an L-shaped relationship between TyG-BMI and the risk of poor self-rated mental health (Figure 2).

**Table 2:** The association of TyG-BMI with self-rated mental health.

TyG-BMI	Total *N*	No. of events (incidence rate^a^)	Crude model		Model 1		Model 2	
			HR (95% CI)	*p* value	HR (95% CI)	*p* value	HR (95% CI)	*p* value
Per 10 units increase								
<204.3	1,476	539 (85.0)	0.92 (0.88–0.96)	<0.001	0.93 (0.89–0.98)	0.003	0.94 (0.90–0.99)	0.014
≥204.3	1,475	487 (75.8)	1.01 (0.98–1.04)	0.600	1.01 (0.98–1.05)	0.506	1.01 (0.97–1.04)	0.717
Per 1 SD increase								
<204.3	1,476	539 (85.0)	0.73 (0.61–0.86)	<0.001	0.77 (0.65–0.92)	0.003	0.80 (0.67–0.96)	0.014
≥204.3	1,475	487 (75.8)	1.03 (0.91–1.18)	0.600	1.04 (0.92–1.19)	0.506	1.03 (0.89–1.18)	0.717

*N*, number; TyG, triglyceride-glucose; HR, hazard ratio; CI, confidence interval; SD, Standard deviation; BMI, body mass index; WHR, waist hip ratio; SBP, systolic blood pressure; DBP, diastolic blood pressure; CKD, chronic kidney disease; LDL-C, low density lipoprotein cholesterol; TC, total cholesterol; HbA1c, glycosylated hemoglobin A1c; FBI, fasting blood insulin.

a Incidence rate was presented as per 1,000 person-years of follow-up.

Model 1: adjusted for sex, age.

Model 2 (Full model): Model1 + further adjusted for, WHR, smoking, drinking, region, marital status, education, occupation, CKD, diabetes mellitus, hypertension, LDL-C, TC, HbA1c, and FBI.

### Subgroup analyses

**Figure 3:**
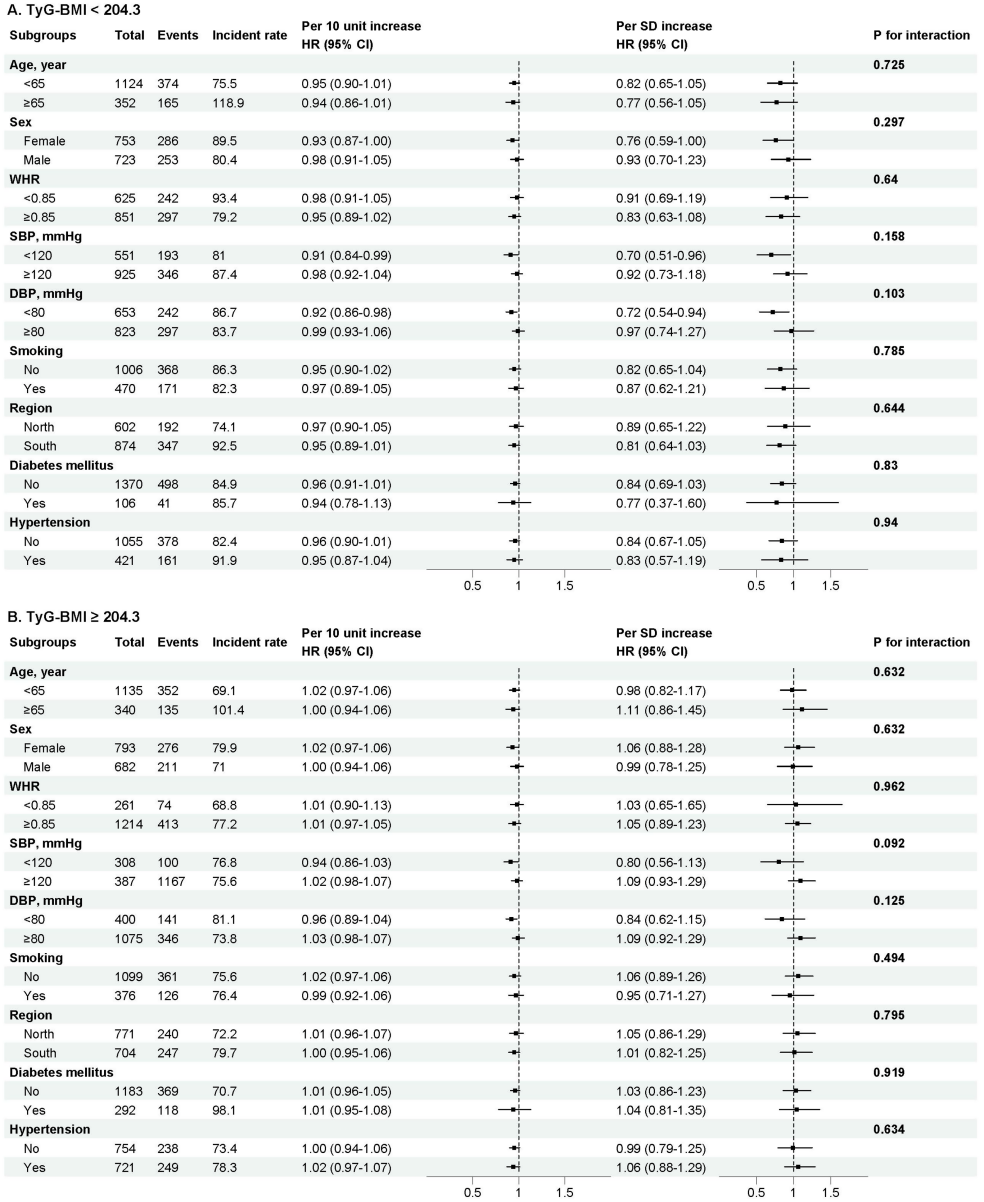
Stratified analyses by potential modifiers of the association between TyG-BMI and self-rated mental health. Forest plot showing the association (expressed as Hazard Ratio, HR, and 95% Confidence Interval) between TyG-BMI and self-rated mental health across various subgroups. Subgroups are defined by age, sex, waist-to-hip ratio (WHR), systolic blood pressure (sbp), diastolic blood pressure (DBP), smoking status, region, and the presence of hypertension or diabetes. The model was adjusted for sex, age, waist-to-hip ratio (WHR), smoking status, drinking status, region, marital status, education level, occupation, chronic kidney disease, hypertension, diabetes mellitus, low-density lipoprotein cholesterol (LDL-C), total cholesterol (TC), glycosylated hemoglobin A1c (HbA1c), and fasting blood insulin (FBI), except where the variable was used as the stratification factor. The *p*-value for interaction tests the homogeneity of the association across subgroups. Incidence rates are presented per 1,000 person-years of follow-up within each subgroup.

Stratified analyses were conducted to further explore the association between exposure and outcome across various subgroups (Figure 3). None of the subgroups—including age, sex, WHR, SBP, DBP, smoking status, geographic region, and the presence of DM or hypertension—significantly modified the relationship (all *p* for interaction >0.05).

### Sensitivity analyses

The results remained consistent when various methods were employed to assess the robustness of the relationship between TyG-BMI and self-rated mental health.

First, the effect of TyG-BMI on self-rated mental health was consistent with the main analysis when using the interval-censoring Cox model. In the group with TyG-BMI < 204.3, both a 10-unit increase in TyG-BMI and a 1 SD increase in TyG-BMI were statistically significant (per 10-unit increase, aHR [95% CI]: 0.94 [0.89–0.99]; per 1 SD increase, aHR [95% CI]: 0.77 [0.62–0.97]). However, no significant association was observed in the TyG-BMI ≥ 204.3 group (Supplementary Table 4). Second, when restricting participants to those with only two follow-up visits, the results remained consistent with the main analysis (Supplementary Table 5). Finally, similar trends were observed between TyG-BMI and self-rated mental health after imputing baseline missing values (Supplementary Table 6).

### Additional analyses

The E-values for the hazard ratio and upper confidence limit for the outcome were 1.61 and 1.20 (Supplementary Figure 2).

## Discussion

In this national, longitudinal cohort study involving 2,951 middle-aged and older Chinese adults with up to 6 years of follow-up, we identified an L-shaped association between TyG-BMI and self-rated mental health after adjusting for potential confounders. This relationship was consistent across various subgroups and sensitivity analyses. The dose–response curve indicated a negative association between TyG-BMI and the risk of poor self-rated mental health up to the inflection point of 204.3. Although the HR per unit change is modest, the clear non-linear relationship and the high prevalence of metabolic dysregulation suggest that TyG-BMI could have meaningful implications for population-level mental health. The present study may provide new insights into the primary prevention of mental health.

Mental health problems are on the rise worldwide (39), particularly among middle-aged and older adults due to population aging. Despite previous studies (7) identifying genetic and environmental factors contributing to mental health disorders, the mechanisms underlying the links between these factors and mental health outcomes remain poorly understood. Numerous observational studies (40, 41) have found that diabetes, IR, and obesity can exacerbate depressive symptoms. In South Korea, a substantial cross-sectional investigation indicated that the risk of depressive symptoms increased by 4% in young people and 17% in non-diabetics with elevated IR (42). Furthermore, several studies have reported a significant association between the TyG index—a surrogate marker of IR—and depression and anxiety (15). Thus, it is critical to identify potential indicators linking the incidence of mental disorders, particularly in relation to alternative metabolic metrics.

Recently, some studies have demonstrated that TyG-BMI is a more robust alternative indicator for the early detection of IR than the TyG index (18). A substantial body of research has indicated a strong correlation between TyG-BMI and various health outcomes, including cardiovascular events, stroke, dementia, non-alcoholic fatty liver disease, pre-diabetes, and diabetes (19–21, 43–45). However, the role of TyG-BMI in the risk of mental disorders has been explored in only a few studies, yielding inconsistent results (23–25). Liu et al. (25) conducted a cross-sectional study using the NHANES database, which showed a positive linear correlation between TyG-BMI and depressive symptoms. In contrast, our study identified an L-shaped non-linear association between TyG-BMI and self-rated mental health. Before the inflection point of 204.3, higher TyG-BMI was significantly associated with a decreased risk of poor self-rated mental health, while this effect was no longer significant when TyG-BMI exceeded 204.3. The heterogeneity in findings between Liu’s study and ours may be partly attributed to differences in study populations, ethnicity, and study design. Consistent with our results, Wang et al. (23) found that not only TyG-BMI but also other metabolic indicators, such as TyG-waist circumference and TyG-waist to height Ratio (WHtR), are valuable in predicting depressive symptoms in both men and women. Notably, a two-part longitudinal study (24) using cluster sampling among college students concluded that high levels of TyG and TyG-BMI were not significantly associated with increased depression and anxiety in young adults.

Moreover, the subgroup analyses revealed that all pre-established variables did not significantly modify the association between TyG-BMI and self-rated mental health, indicating that our results are applicable to the majority of the general population. The consistency of this association across subgroups not only suggests its stability but also warrants consideration of alternative explanations, such as the broad, non-specific nature of the relationship or the potential influence of unmeasured confounding factors. Furthermore, it is important to address an apparent discrepancy in the results: the crude incidence of poor self-rated mental health was higher in the lower TyG-BMI group. This initially counterintuitive finding is likely attributable to substantial confounding by sociodemographic factors. The emergence of a significant inverse association after full adjustment underscores the critical importance of considering sociodemographic context when interpreting the relationship between metabolic health and mental well-being, and it confirms that the adjusted models provide a more valid estimate of the independent relationship. Overall, our study suggests that TyG-BMI could serve as a novel potential biomarker for self-rated mental health. Our findings indicate that primary interventions aimed at improving metabolic health may also benefit psychological well-being, particularly in older adults. Further studies are needed to confirm our results and to explore the underlying mechanisms.

The current data do not elucidate the causal mechanisms underlying the association between TyG-BMI and self-rated mental health. However, the relationship may be potentially linked to BMI or IR due to their close association. The impact of overweight on mental health remains controversial. A positive correlation between obesity and impaired mental health has been identified in numerous studies (46, 47), while opposing views have been reported in a few studies (48, 49). Notably, the Canadian CaMos study involving 9,423 adults found that being classified as overweight was associated with a slightly better health-related quality of life (HRQOL) in men (50). Additionally, a study of 43,534 Dutch adults (51) revealed a U-shaped curve between BMI and depression, with the lowest levels of depressive symptoms observed in the overweight category. Furthermore, Linna et al. (52) reported a U-shaped association between BMI and mental disorders, estimating the optimal BMI for mental health in men to range from 26.1 to 28.9 kg/m^2^. Consistent with these findings, our study suggests that the association between TyG-BMI and self-rated mental health may also be L-shaped, with both very low levels of BMI potentially contributing to impaired mental health. This resembles the relationship between BMI and mortality, with some studies suggesting that being overweight is a protective factor against mortality in middle-aged and older populations (53, 54). Importantly, an abnormally high TyG-BMI or BMI could lead to inflammation and dysregulation of the hypothalamic–pituitary–adrenal (HPA) axis, potentially inducing the development of mental disorders (55). The exact causal mechanisms linking TyG-BMI and mental health are likely more complex and warrant further investigation.

The major strengths of this study include that it provides several novel insights that distinguish it from previous analyses. First, to our knowledge, this is the first national study in China with a prospective design investigating the relationship between TyG-BMI and self-rated mental health among middle-aged and older participants. Second, our focus on self-rated mental health, a holistic measure of subjective well-being, complements the existing literature on clinical diagnoses and highlights the importance of metabolic health for overall psychological well-being in the general population.

Certain limitations should also be acknowledged. First, the analysis was restricted to three waves of the CHNS (2009, 2011, and 2015) as these were the only cycles with the necessary laboratory data for calculating TyG-BMI. This design choice, while essential for ensuring the accuracy of our primary exposure variable, resulted in a shorter follow-up duration with fewer assessment points compared to the full cohort span. Although the retained sample size remains substantial and provided sufficient power to detect the reported non-linear association, this structure may have limited our ability to capture longer-term mental health dynamics and reduced statistical power for certain longitudinal or time-sensitive analyses. Second, although we adjusted for potential confounders such as demographics, lifestyle factor, and blood biochemical markers as much as possible, the E-values for the hazard ratio and the lower confidence limit for the primary outcome were relatively small, indicating that minimal unmeasured confounding could diminish the observed association or its 95% confidence interval to the null. Thus, residual confounding such as socioeconomic status, detailed dietary patterns, and family history of psychiatric disorders cannot be completely ruled out. In addition, the use of a single-item, self-rated mental health measure has not been formally validated, and the lack of established psychometric properties means its ability to accurately capture the intended construct of overall mental well-being is uncertain, making it subject to reporting heterogeneity. Third, given the observational nature of the study, we cannot establish a causal relationship between TyG-BMI and self-rated mental health. Fourth, considering that death represents an inevitable competing risk, we might underestimate the relationship between TyG-BMI and self-rated mental health. However, in our study population, only 74 (2.5%) participants died during the follow-up, which is unlikely to significantly alter the trend of the results. Furthermore, it is important to note that this specific threshold, 204.3 of TYG-BMI, was derived empirically from our data using restricted cubic spline analysis. Its generalizability and precise biological meaning require confirmation through further mechanistic investigations and validation in diverse, independent populations. Finally, this study was limited to the Chinese general population, and the study population primarily consisted of older adults. Future studies should aim to include younger age groups and diverse ethnic cohorts to validate whether the observed association between TyG-BMI and self-rated mental health is consistent across different age groups and national contexts.

## Conclusion

We identified an L-shaped non-linear relationship within a longitudinal national cohort, showing that TyG-BMI levels below the inflection point of 204.3 were associated with a lower risk of poor self-rated mental health. These findings suggest that maintaining TyG-BMI levels within the optimal range may be associated with a reduced risk of mental health concerns. However, it is important to note that these are observational associations. In clinical practice, TyG-BMI is a readily accessible indicator that may aid in risk stratification, providing clinicians with a tool to offer more personalized prevention and treatment strategies within the general population.

## Data Availability

The datasets presented in this study can be found in online repositories. The names of the repository/repositories and accession number(s) can be found in the article/Supplementary material.

## Footnotes

^1^ http://www.cpc.unc.edu/projects/china

^2^ http://www.r-project.org/

## Glossary

WHO: World Health Organization
IR: Insulin resistance
TyG index: Triglyceride-glucose index
TG: Triglycerides
FBG: Fasting blood glucose
BMI: Body mass index
TyG-BMI: Triglyceride glucose-body mass index
CHNS: China Health and Nutrition Survey
STROBE: Strengthening the Reporting of Observational Studies in Epidemiology
TC: Total cholesterol
HDL-C: High density lipoprotein cholesterol
LDL-C: Low density lipoprotein cholesterol
HbA1c: Glycosylated hemoglobin
BUN: Blood urea nitrogen
SCr: Serum creatinine
hsCRP: High sensitivity C reactive protein
FBI: Fasting blood insulin
WHR: Waist to hip ratio
WHtR: waist to height ratio
SBP: Systolic blood pressure
DBP: Diastolic blood pressure
DM: Diabetes mellitus
ADA: American Diabetes Association
CKD: Chronic kidney disease
eGFR: Estimated glomerular filtration rate
CKD-EPI: Chronic Kidney Disease Epidemiology Collaboration equation
SD: Standard deviation
HR: Hazard ratio
aHR: Adjusted hazard ratio
CI: Confidence intervals
VIF: Variance inflation factor
RCS: Restricted cubic spline
