# Supplementary material for: L-shaped association of triglyceride glucose-body mass index and self-rated mental health among the middle-aged and older adults: a national cohort study in China
Source: Front Public Health. 2025 Nov 25;13:1672881. doi: 10.3389/fpubh.2025.1672881 (PMC12685690; doi:10.3389/fpubh.2025.1672881)
Supplement: Supplementary file 3 [file Supplementary_file_1.docx]

**Supplementary Material Content**

**Supplemental Tables**

**Supplemental Table 1.** Distribution of missing variables.

**Supplemental Table 2.** Baseline characteristics of excluded and included participants.

**Supplemental Table 3.** Baseline characteristics of study participants stratified by self-rated mental health.

**Supplemental Table 4.** The association of TyG-BMI with self-rated mental health using interval censored Cox regression model.

**Supplemental Table 5.** The association of TyG-BMI with self-rated mental health in participants limited with two follow-up visits.

**Supplementary Table 6.** The association of TyG-BMI with self-rated mental health after imputing the baseline missing values.

**Supplemental Figures**

**Supplemental Figure 1.** Assessment of Multicollinearity Among Predictors Using Variance Inflation Factors (VIF). This figure presents the variance inflation factor (VIF) for each predictor variable included in the multivariable model. A VIF value of less than 5 was considered to indicate no significant multicollinearity, suggesting that the predictors are sufficiently independent for reliable effect estimation. The results demonstrate that multicollinearity was not a major concern in our analysis.

**Abbreviations**: TC, total cholesterol; LDL-C, low density lipoprotein cholesterol; HbA1c, glycosylated hemoglobin A1c; DM, diabetes mellitus; CKD, chronic kidney disease; WHR, waist hip ratio; FBI, fasting blood insulin.

**Supplemental Figure** **2**. E-value analysis to assess the extent of unmeasured confounding that would be required to negate the observed results. This figure summarizes the E-value analysis, which quantifies the minimum strength of association that an unmeasured confounder would need to have with both the exposure (TyG-BMI) and the outcome to fully explain away the observed association. The E-values for the point estimate and the confidence interval limit are presented, indicating the robustness of the results to potential unmeasured confounding.

**Abbreviations**: RR, Risk Ratio; E, exposure; D, outcome; U, unmeasured confounder.

**Supplemental Table 1.** Distribution of missing variables.

| Characteristic | Number of participants^*^ (N=87) |
| --- | --- |
| SBP | 1 |
| DBP | 1 |
| WHR | 52 |
| HbA1c | 24 |
| FBI | 7 |
| LDL-C | 1 |
| Smoking status | 2 |
| Education | 4 |

***** Some participants displayed multiple missing variables.

**Abbreviations**: SBP, systolic blood pressure; DBP, diastolic blood pressure; WHR, waist hip ratio; HbA1c, hemoglobin A1c; FBI, fasting blood insulin; LDL-C, low density lipoprotein cholesterol.

**Supplemental Table 2.** Baseline characteristics of excluded and included participants.

| **Characteristics** | **Overall (N=5553)** | **Included (N=2951)** | **Excluded (N=2602)** | ***P* value** |
| --- | --- | --- | --- | --- |
| TyG-BMI | 202.96 [177.60, 231.24] | 204.30 [179.58, 231.80] | 201.56 [175.18, 230.33] | 0.005 |
| TyG index | 8.60 [8.20, 9.10] | 8.70 [8.20, 9.10] | 8.60 [8.20, 9.10] | 0.301 |
| BMI, kg/m² | 23.40 [21.20, 25.80] | 23.60 [21.40, 25.80] | 23.20 [20.90, 25.80] | 0.004 |
| Age, years | 58.00 [52.00, 66.00] | 56.00 [51.00, 64.00] | 59.00 [53.00, 69.00] | <0.001 |
| Male (%) | 2605 (46.9) | 1405 (47.6) | 1200 (46.1) | 0.278 |
| SBP, mmHg | 128.00 [118.00, 140.00] | 127.00 [118.00, 140.00] | 129.00 [119.00, 141.00] | 0.001 |
| DBP, mmHg | 81.00 [76.00, 90.00] | 81.00 [76.00, 89.00] | 81.00 [75.00, 90.00] | 0.998 |
| WHR | 0.90 [0.80, 0.90] | 0.90 [0.80, 0.90] | 0.90 [0.80, 0.90] | 0.915 |
| Smoking, n (%) | 1558 (28.1) | 846 (28.7) | 712 (27.5) | 0.369 |
| Drinking, n (%) | 1751 (31.6) | 983 (33.3) | 768 (29.7) | 0.004 |
| Region^*^, n (%) |  |  |  | <0.001 |
| North | 2347 (42.3) | 1373 (46.5) | 974 (37.4) |  |
| South | 3206 (57.7) | 1578 (53.5) | 1628 (62.6) |  |
| Education, n (%) |  |  |  | <0.001 |
| Illiteracy | 1853 (33.5) | 829 (28.1) | 1024 (39.8) |  |
| Primary school | 1150 (20.8) | 601 (20.4) | 549 (21.3) |  |
| Middle school | 2053 (37.1) | 1224 (41.5) | 829 (32.2) |  |
| High school or above | 471 (8.5) | 297 (10.1) | 174 (6.8) |  |
| Occupation, n (%) |  |  |  | <0.001 |
| Farmer | 1511 (27.3) | 808 (27.4) | 703 (27.2) |  |
| Worker | 166 (3.0) | 99 (3.4) | 67 (2.6) |  |
| Unemployed | 2894 (52.3) | 1445 (49.0) | 1449 (56.1) |  |
| Others | 960 (17.4) | 594 (20.2) | 366 (14.2) |  |
| Marital status |  |  |  | <0.001 |
| Married | 45 (0.8) | 14 (0.5) | 31 (1.2) |  |
| Unmarried | 4741 (85.6) | 2648 (89.7) | 2093 (80.9) |  |
| Widowed | 656 (11.8) | 243 (8.2) | 413 (16.0) |  |
| Others | 97 (1.8) | 46 (1.6) | 51 (2.0) |  |
| CKD, n (%) | 937 (16.9) | 396 (13.4) | 541 (21.0) | <0.001 |
| Diabetes mellitus, n (%) | 801 (14.4) | 398 (13.5) | 403 (15.5) | 0.059 |
| Hypertension | 2264 (41.2) | 1142 (38.7) | 1122 (44.2) | <0.001 |
| Laboratory data |  |  |  |  |
| Triglycerides, mmol/L | 1.34 [0.91, 2.05] | 1.34 [0.92, 2.06] | 1.32 [0.90, 2.04] | 0.345 |
| TC, mmol/L | 4.96 [4.35, 5.65] | 4.95 [4.35, 5.65] | 4.96 [4.36, 5.65] | 0.850 |
| LDL-C, mmol/L | 3.06 [2.48, 3.69] | 3.07 [2.49, 3.69] | 3.05 [2.48, 3.68] | 0.626 |
| HDL-C, mmol/L | 1.40 [1.17, 1.66] | 1.40 [1.17, 1.66] | 1.40 [1.17, 1.66] | 0.998 |
| HbA1c, % | 5.60 [5.30, 6.00] | 5.60 [5.30, 6.00] | 5.60 [5.30, 6.00] | 0.568 |
| FBG, mmol/L | 5.25 [4.81, 5.79] | 5.26 [4.82, 5.77] | 5.22 [4.79, 5.81] | 0.483 |
| FBI, mmol/L | 10.35 [7.23, 15.13] | 10.32 [7.26, 14.96] | 10.36 [7.20, 15.33] | 0.641 |
| eGFR, ml/min/1.73m² | 73.00 [64.00, 83.00] | 75.00 [66.00, 84.00] | 72.00 [62.00, 81.00] | <0.001 |

^*^Region was divided into north (Heilongjiang, Liaoning, Beijing, Shandong, and Henan), and south (Jiangsu, Shanghai, Hubei, Hunan, Chongqing, Guizhou, and Guangxi) based on the Qinling Mountains-Huaihe River Line.

**Abbreviations**: N, number; TyG, triglyceride-glucose; SBP, systolic blood pressure; DBP, diastolic blood pressure; BMI, body mass index; WHR, waist hip ratio; CKD, chronic kidney disease; TC, total cholesterol; LDL-C, low density lipoprotein cholesterol; HDL-C, high density lipoprotein cholesterol; HbA1c, glycosylated hemoglobin A1c; FBG, fasting blood glucose; FBI, fasting blood insulin; eGFR, estimated glomerular filtration rate.

**Supplemental Table 3.** Baseline characteristics of study participants stratified by self-rated mental health.

| **Characteristics** | **Overall (N=2951)** | **Mental health** | | ***P* value** |
| --- | --- | --- | --- | --- |
|  |  | **No (N=1925)** | **Yes (N=1026)** |  |
| TyG-BMI | 204.30 [179.58, 231.80] | 205.66 [181.80, 232.18] | 201.60 [175.29, 229.59] | 0.006 |
| TyG index | 8.70 [8.20, 9.10] | 8.70 [8.20, 9.10] | 8.60 [8.20, 9.20] | 0.541 |
| BMI, kg/m² | 23.60 [21.40, 25.80] | 23.70 [21.60, 25.90] | 23.30 [21.00, 25.60] | 0.001 |
| Age, years | 56.00 [51.00, 64.00] | 56.00 [50.00, 63.00] | 59.00 [52.00, 66.00] | <0.001 |
| Male (%) | 1405 (47.6) | 941 (48.9) | 464 (45.2) | 0.063 |
| SBP, mmHg | 127.00 [118.00, 140.00] | 126.00 [118.00, 139.00] | 128.00 [118.00, 141.00] | 0.019 |
| DBP, mmHg | 81.00 [76.00, 89.00] | 81.00 [77.00, 89.00] | 81.00 [75.00, 90.00] | 0.750 |
| WHR | 0.90 [0.80, 0.90] | 0.90 [0.80, 0.90] | 0.90 [0.80, 0.90] | 0.560 |
| Smoking, n (%) | 846 (28.7) | 549 (28.5) | 297 (28.9) | 0.840 |
| Drinking, n (%) | 983 (33.3) | 680 (35.3) | 303 (29.5) | 0.002 |
| Region^*^, n (%) |  |  |  | 0.001 |
| North | 1373 (46.5) | 941 (48.9) | 432 (42.1) |  |
| South | 1578 (53.5) | 984 (51.1) | 594 (57.9) |  |
| Education, n (%) |  |  |  | <0.001 |
| Illiteracy | 829 (28.1) | 469 (24.4) | 360 (35.1) |  |
| Primary school | 601 (20.4) | 380 (19.7) | 221 (21.5) |  |
| Middle school | 1224 (41.5) | 847 (44.0) | 377 (36.7) |  |
| High school or above | 297 (10.1) | 229 (11.9) | 68 (6.6) |  |
| Occupation, n (%) |  |  |  | <0.001 |
| Farmer | 808 (27.4) | 492 (25.6) | 316 (30.9) |  |
| Worker | 99 (3.4) | 55 (2.9) | 44 (4.3) |  |
| Unemployed | 1445 (49.0) | 935 (48.6) | 510 (49.9) |  |
| Others | 594 (20.2) | 442 (23.0) | 152 (14.9) |  |
| Marital status |  |  |  | 0.005 |
| Married | 14 (0.5) | 9 (0.5) | 5 (0.5) |  |
| Unmarried | 2648 (89.7) | 1755 (91.2) | 893 (87.0) |  |
| Widowed | 243 (8.2) | 136 (7.1) | 107 (10.4) |  |
| Others | 46 (1.6) | 25 (1.3) | 21 (2.0) |  |
| CKD, n (%) | 396 (13.4) | 245 (12.7) | 151 (14.7) | 0.146 |
| Diabetes mellitus, n (%) | 398 (13.5) | 239 (12.4) | 159 (15.5) | 0.023 |
| Hypertension | 1142 (38.7) | 732 (38.0) | 410 (40.0) | 0.323 |
| Laboratory data |  |  |  |  |
| Triglycerides, mmol/L | 1.34 [0.92, 2.06] | 1.36 [0.92, 2.06] | 1.31 [0.90, 2.08] | 0.389 |
| TC, mmol/L | 4.95 [4.35, 5.65] | 4.96 [4.37, 5.64] | 4.92 [4.32, 5.65] | 0.489 |
| LDL-C, mmol/L | 3.07 [2.49, 3.69] | 3.09 [2.52, 3.69] | 3.04 [2.47, 3.70] | 0.522 |
| HDL-C, mmol/L | 1.40 [1.17, 1.66] | 1.39 [1.17, 1.66] | 1.41 [1.17, 1.66] | 0.745 |
| HbA1c, % | 5.60 [5.30, 6.00] | 5.60 [5.30, 6.00] | 5.60 [5.30, 5.90] | 0.500 |
| FBG, mmol/L | 5.26 [4.82, 5.77] | 5.27 [4.83, 5.74] | 5.26 [4.80, 5.83] | 0.919 |
| FBI, mmol/L | 10.32 [7.26, 14.96] | 10.48 [7.41, 15.03] | 9.99 [7.01, 14.81] | 0.122 |
| eGFR, ml/min/1.73m² | 75.00 [66.00, 84.00] | 75.00 [66.00, 85.00] | 74.00 [65.00, 84.00] | 0.082 |

^*^Region was divided into north (Heilongjiang, Liaoning, Beijing, Shandong, and Henan), and south (Jiangsu, Shanghai, Hubei, Hunan, Chongqing, Guizhou, and Guangxi) based on the Qinling Mountains-Huaihe River Line.

**Abbreviations**: N, number; TyG, triglyceride-glucose; SBP, systolic blood pressure; DBP, diastolic blood pressure; BMI, body mass index; WHR, waist hip ratio; CKD, chronic kidney disease; TC, total cholesterol; LDL-C, low density lipoprotein cholesterol; HDL-C, high density lipoprotein cholesterol; HbA1c, glycosylated hemoglobin A1c; FBG, fasting blood glucose; FBI, fasting blood insulin; eGFR, estimated glomerular filtration rate.

**Supplemental Table 4.** The association of TyG-BMI with self-rated mental health using interval censored Cox regression model.

| TyG-BMI | Total  N | No. of Events  (incidence rate^a^) | **Crude model** | | **Model 1** | | **Model 2** | |
| --- | --- | --- | --- | --- | --- | --- | --- | --- |
|  |  |  | HR (95% CI) | *P* value | HR (95% CI) | *P* value | HR (95% CI) | *P* value |
| **Per 10 units increase** |  |  |  |  |  |  |  |  |
| **<204.3** | 1476 | 539 (85.0) | 0.91 (0.87-0.96) | <0.001 | 0.93 (0.88-0.97) | 0.002 | 0.94 (0.89-0.99) | 0.002 |
| **≥204.3** | 1475 | 487 (75.8) | 1.00 (0.97-1.04) | 0.812 | 1.01 (0.97-1.04) | 0.638 | 1.00 (0.97-1.04) | 0.813 |
| **Per 1 SD increase** |  |  |  |  |  |  |  |  |
| **<204.3** | 1476 | 539 (85.0) | 0.69 (0.57-0.84) | <0.001 | 0.74 (0.61-0.90) | 0.002 | 0.77 (0.62-0.97) | 0.002 |
| **≥204.3** | 1475 | 487 (75.8) | 1.02 (0.88-1.17) | 0.812 | 1.03 (0.90-1.18) | 0.638 | 1.02 (0.88-1.17) | 0.813 |

**Abbreviations**: N, number; TyG, triglyceride-glucose; HR, hazard ratio; CI, confidence interval; SD, Standard deviation; BMI, body mass index; WHR, waist hip ratio; SBP, systolic blood pressure; DBP, diastolic blood pressure; CKD, chronic kidney disease; LDL-C, low density lipoprotein cholesterol; TC, total cholesterol; HbA1c, glycosylated hemoglobin A1c; FBI, fasting blood insulin.

^a^Incidence rate was presented as per 1000 person-years of follow-up.

**Model1**: adjusted for sex, age.

**Model2 (Full model)**: Model1+further adjusted for, WHR, smoking, drinking, region, marital status, education, occupation, CKD, diabetes mellitus, hypertension, LDL-C, TC, HbA1c, and FBI.

**Supplemental Table 5.** The association of TyG-BMI with self-rated mental health in participants limited with two follow-up visits.

| TyG-BMI | Total  N | No. of Events  (incidence rate^a^) | **Crude model** | | **Model 1** | | **Model 2** | |
| --- | --- | --- | --- | --- | --- | --- | --- | --- |
|  |  |  | HR (95% CI) | *P* value | HR (95% CI) | *P* value | HR (95% CI) | *P* value |
| **Per 10 units increase** |  |  |  |  |  |  |  |  |
| **<204.3** | 1208 | 476 (83.2) | 0.90 (0.86-0.95) | <0.001 | 0.91 (0.87-0.96) | <0.001 | 0.92 (0.88-0.97) | 0.002 |
| **≥204.3** | 1188 | 427 (74.5) | 1.00 (0.96-1.03) | 0.827 | 1.00 (0.96-1.04) | 0.892 | 0.99 (0.95-1.03) | 0.734 |
| **Per 1 SD increase** |  |  |  |  |  |  |  |  |
| **<204.3** | 1208 | 476 (83.2) | 0.66 (0.55-0.81) | <0.001 | 0.70 (0.58-0.85) | <0.001 | 0.73 (0.59-0.89) | 0.002 |
| **≥204.3** | 1188 | 427 (74.5) | 0.98 (0.85-1.14) | 0.827 | 0.99 (0.85-1.15) | 0.892 | 0.97 (0.83-1.14) | 0.734 |

**Abbreviations**: N, number; TyG, triglyceride-glucose; HR, hazard ratio; CI, confidence interval; SD, Standard deviation; BMI, body mass index; WHR, waist hip ratio; SBP, systolic blood pressure; DBP, diastolic blood pressure; CKD, chronic kidney disease; LDL-C, low density lipoprotein cholesterol; TC, total cholesterol; HbA1c, glycosylated hemoglobin A1c; FBI, fasting blood insulin.

^a^Incidence rate was presented as per 1000 person-years of follow-up.

**Model1**: adjusted for sex, age.

**Model2 (Full model)**: Model1+further adjusted for, WHR, smoking, drinking, region, marital status, education, occupation, CKD, diabetes mellitus, hypertension, LDL-C, TC, HbA1c, and FBI.

**Supplementary Table 6.** The association of TyG-BMI with self-rated mental health after imputing the baseline missing values.

| TyG-BMI | Total  N | No. of Events  (incidence rate^a^) | **Crude model** | | **Model 1** | | **Model 2** | |
| --- | --- | --- | --- | --- | --- | --- | --- | --- |
|  |  |  | HR (95% CI) | *P* value | HR (95% CI) | *P* value | HR (95% CI) | *P* value |
| **Per 10 units increase** |  |  |  |  |  |  |  |  |
| **<204.3** | 1505 | 549 (85.1) | 0.92 (0.88-0.96) | <0.001 | 0.94 (0.89-0.98) | 0.004 | 0.94 (0.90-0.99) | 0.016 |
| **≥204.3** | 1533 | 505 (75.9) | 1.01 (0.97-1.04) | 0.713 | 1.01 (0.98-1.04) | 0.597 | 1.01 (0.97-1.04) | 0.770 |
| **Per 1 SD increase** |  |  |  |  |  |  |  |  |
| **<204.3** | 1476 | 539 (85.0) | 0.72 (0.60-0.86) | <0.001 | 0.77 (0.64-0.92) | 0.004 | 0.79 (0.66-0.96) | 0.016 |
| **≥204.3** | 1475 | 487 (75.8) | 1.02 (0.90-1.17) | 0.713 | 1.04 (0.91-1.18) | 0.597 | 1.02 (0.89-1.17) | 0.770 |

**Abbreviations**: N, number; TyG, triglyceride-glucose; HR, hazard ratio; CI, confidence interval; SD, Standard deviation; BMI, body mass index; WHR, waist hip ratio; SBP, systolic blood pressure; DBP, diastolic blood pressure; CKD, chronic kidney disease; LDL-C, low density lipoprotein cholesterol; TC, total cholesterol; HbA1c, glycosylated hemoglobin A1c; FBI, fasting blood insulin.

^a^Incidence rate was presented as per 1000 person-years of follow-up.

**Model1**: adjusted for sex, age.

**Model2 (Full model)**: Model1+further adjusted for, WHR, smoking, drinking, region, marital status, education, occupation, CKD, diabetes mellitus, hypertension, LDL-C, TC, HbA1c, and FBI.
